# Associations among perfluorooctanesulfonic/perfluorooctanoic acid levels, nuclear receptor gene polymorphisms, and lipid levels in pregnant women in the Hokkaido study

**DOI:** 10.1038/s41598-021-89285-2

**Published:** 2021-05-11

**Authors:** Sumitaka Kobayashi, Fumihiro Sata, Houman Goudarzi, Atsuko Araki, Chihiro Miyashita, Seiko Sasaki, Emiko Okada, Yusuke Iwasaki, Tamie Nakajima, Reiko Kishi

**Affiliations:** 1 Center for Environmental and Health Sciences, Hokkaido University, North-12, West-7, Kita-ku, Sapporo, 060-0812 Japan; 2 Health Center, Chuo University, Tokyo, Japan; 3 Department of Respiratory Medicine, Faculty of Medicine and Graduate School of Medicine, Hokkaido University, Sapporo, Japan; 4 Department of Public Health, Faculty of Medicine and Graduate School of Medicine, Hokkaido University, Sapporo, Japan; 5 Department of Nutritional Epidemiology and Shokuiku, National Institutes of Biomedical Innovation, Health, and Nutrition, Tokyo, Japan; 6 Department of Biopharmaceutics and Analytical Science, Hoshi University, Tokyo, Japan; 7 College of Life and Health Sciences, Chubu University, Kasugai, Japan

**Keywords:** Environmental sciences, Risk factors

## Abstract

The effect of interactions between perfluorooctanesulfonic (PFOS)/perfluorooctanoic acid (PFOA) levels and nuclear receptor genotypes on fatty acid (FA) levels, including those of triglycerides, is not clear understood. Therefore, in the present study, we aimed to analyse the association of PFOS/PFOA levels and single-nucleotide polymorphisms (SNPs) in nuclear receptors with FA levels in pregnant women. We analysed 504 mothers in a birth cohort between 2002 and 2005 in Japan. Serum PFOS/PFOA and FA levels were measured using liquid chromatography-tandem mass spectrometry and gas chromatography-mass spectrometry. Maternal genotypes in *PPARA* (rs1800234; rs135561), *PPARG* (rs3856806), *PPARGC1A* (rs2970847; rs8192678), *PPARD* (rs1053049; rs2267668), *CAR* (rs2307424; rs2501873), *LXRA* (rs2279238) and *LXRB* (rs1405655; rs2303044; rs4802703) were analysed. When gene-environment interaction was considered, PFOS exposure (log_10_ scale) decreased palmitic, palmitoleic, and oleic acid levels (log_10_ scale), with the observed β in the range of − 0.452 to − 0.244; *PPARGC1A* (rs8192678) and *PPARD* (rs1053049; rs2267668) genotypes decreased triglyceride, palmitic, palmitoleic, and oleic acid levels, with the observed β in the range of − 0.266 to − 0.176. Interactions between PFOS exposure and SNPs were significant for palmitic acid (*P*_*int*_ = 0.004 to 0.017). In conclusion, the interactions between maternal PFOS levels and *PPARGC1A* or *PPARD* may modify maternal FA levels.

## Introduction

The genetic makeup of a person and the environmental factors might be responsible for regulating the levels of serum lipids, such as fatty acids (FA) and triglycerides (TG)^1^. Previous epidemiological studies have identified some well-defined gene–environment interactions supporting the concept that environment factors, such as perfluoroalkyl acids in association with the individual’s genotype, particularly nuclear receptors, might determine health outcomes^2–8^. Since maternal genetic factors, together with environmental factors, may influence maternal lipid levels, it is important to examine the gene-environment interactions that affect maternal lipid levels.

Perfluorooctanesulfonic acid (PFOS) and perfluorooctanoic acid (PFOA) have been used for decades in several industrial and chemical applications as processing aids in impregnation agents for use in textiles, carpets, and paper. In humans, diet is considered a common source of exposure to PFOS and PFOA. In animals, one of the main adverse health effects of PFOS and PFOA is reproductive toxicity. PFOS and PFOA can adversely affect the health of human offspring. Recently, we reported that maternal PFOS and PFOA levels were associated with reduced birth size^9,10^ and the risk of infectious/allergic diseases in childhood^11–13^. Maternal PFOS/PFOA levels during pregnancy were also associated with triglyceride (TG) and fatty acid (FA) levels in maternal blood samples^14,15^, the affected mothers’ offspring^16^, and an increased prevalence of overweight female offspring at 20 years of age^17^. FA components, which include palmitic acid, stearic acid, palmitoleic acid, oleic acid, linoleic acid, α-linolenic acid, arachidonic acid, eicosapentaenoic acid (EPA), and docosahexaenoic acid (DHA), constitute a major fraction of TGs^18^. Hence, it is important to monitor both TG and FA levels from the foetal period to adulthood to determine the effect of maternal PFOS/PFOA exposure during pregnancy.

Increased PFOS or PFOA levels were associated with decreased TG levels in our previous study^18^ and another study conducted in Spain^19^. However, increased PFOS or PFOA levels were not associated with decreased TG levels in a previous Norwegian study^19^. Given these conflicting results, associations of maternal PFOS and PFOA levels with TG and FA remain unclear. The inconsistent results might be due to the binding of PFOS and PFOA to nuclear receptors. Receptors are important molecules that convey information within cells by sensing stimuli from the outside. PFOS and PFOA bind to the peroxisome proliferator-activated receptors (PPARs), constitutive androstane receptors (CARs), and liver X receptors (LXRs) in human and rodent hepatocytes^20^. In our previous studies, we observed gene-environment interactions between child growth and dioxin levels in pregnant women, as well as smoking exposure^6–8^. However, no previous study had reported the interactions between maternal genetic polymorphisms in genes encoding *PPARs*, *CARs*, or *LXRs* and the effects of PFOS/PFOA exposure on TG/FA levels.

PPARs, CARs, and LXRs are involved in lipid homeostasis^21–23^. Genes encoded in PPARs, CARs, and LXRs include several single nucleotide polymorphisms (SNPs), which are associated with disease susceptibility. These SNPs include receptor genetic polymorphisms such as *PPAR alpha* (*PPARA*) (T>C, Val227Ala; rs1800234; exon 6)^24^, *PPARA* (G>A, rs135561; promotor region)^25^, *PPAR delta* (*PPARD*) (T>C, rs1053049; 3′-untranslated region)^26^, *PPARD* (A>G, rs2267668; exon 3)[ 26], *PPAR gamma* (*PPARG*) (C>T, His449His; rs3856806; exon 6)^27^, *PPARG coactivator 1-alpha* (*PPARGC1A*; C>T, Thr394Thr; rs2970847; exon 8)^28^, *PPARGC1A* (G>A, rs8192678; exon 8)^29^, *CAR* (T>C, Pro180Pro; rs2307424; exon 5)^30^, *CAR* (A>G, rs2501873; intron 3)^31^, *LXR alpha* (*LXRA*) (C>T, Ser99Ser; rs2279238; exon 3)^32,33^, *LXR beta* (*LXRB*) (T>C, rs1405655; intron 7)^34,35^, *LXRB* (G>A, rs2303044; intron 8)^36^, and *LXRB* (G>A; rs4802703; intron 8)^36^. To date, limited information is available regarding the association between these genetic polymorphisms and lipid homeostasis. The receptors encoded by these genes are known to interact with PFOS and PFOA. Therefore, it is possible that SNPs in these genes could contribute to the maintenance of lipid homeostasis. Further, it is predicted that not only genetic polymorphisms of disease susceptible genes but also genetic polymorphisms in receptors can contribute to changes in lipid levels.

Therefore, we first targeted and selected three genes, *PPAR*, *CAR*, and *LXR*, which are orphan nuclear receptors that are expected to affect FA levels and are activated by exposure to PFOS and PFOA. The SNPs located in potentially functional regions (mainly coding and promoter regions) were given priority. Next, among these genes, we selected 13 SNPs, which are reportedly associated with disease susceptibilities to cancer, nonalcoholic fatty acid disease, type 2 diabetes mellitus, and obesity, using database SNP (dbSNP) of the National Center for Biological Information (NCBI). All 13 SNPs had a minor allele frequency of more than 5% and were included for subsequent genotyping. A 5% or more frequency of the minor alleles among pregnant Japanese women is necessary to secure statistical powers for examining the health outcomes.

In the Hokkaido Study on Environment and Children’s Health, we examined the association between serum PFOS and PFOA levels and TG and FA (palmitic acid, palmitoleic acid, stearic acid, oleic acid, linoleic acid, α-linolenic acid, arachidonic acid, EPA, and DHA) levels in maternal serum among pregnant Japanese women^14^. In this follow-up study, we examined associations between indicated serum markers and the above-mentioned 13 SNPs in the nuclear receptor genes.

## Results

### Maternal characteristics

Table 1 shows the characteristics of the mothers in the study. Mean maternal age and pre-pregnancy body mass index (BMI) were 30.4 years and 21.2 kg/m^2^, respectively. Among participants, 18.3% were smokers in the third trimester of pregnancy, and 30.6% were alcohol drinkers during pregnancy. Medians of PFOS, PFOA, and triglyceride levels in maternal serum during pregnancy were 5.4 ng/mL, 1.4 ng/mL, and 80.2 mg/100 mL, respectively.

**Table 1:** Maternal characteristics (n = 504).

Characteristics	Mean ± SD, n (%), or median (IQR)
**Basic characteristics**	
Age (years)^a^	30.4 ± 4.9
Pre-pregnancy BMI (kg/m^2^)^a^	21.2 ± 3.2
Parity (primiparous)^b^	240 (47.6)
Smoking in the 3rd trimester (yes)^b^	92 (18.3)
Alcohol consumption during pregnancy (yes)^b^	154 (30.6)
Annual household income (≥ 5 million Japanese yen)^b^	152 (30.2)
**Maternal serum levels and sampling period**	
PFOS (ng/mL)^c^	5.4 (4.0, 7.4)
PFOA (ng/mL)^c^	1.4 (0.9, 2.0)
Triglyceride (mg/100 mL)^c^	80.2 (9.8, 447.5)
Palmitic acid (μg/mL)^c^	1,875.9 (1,506.6, 2,410.2)
Palmitoleic acid (μg/mL)^c^	101.9 (75.5, 149.6)
Stearic acid (μg/mL)^c^	524.0 (427.5, 621.8)
Oleic acid (μg/mL)^c^	1,098.5 (844.8, 1,405.5)
Linoleic acid (μg/mL)^c^	688.6 (478.2, 917.0)
α-linolenic acid (μg/mL)^c^	9.6 (5.0, 14.6)
Arachidonic acid (μg/mL)^c^	63.9 (43.7, 93.5)
EPA (μg/mL)^c^	8.4 (4.0, 13.4)
DHA (μg/mL)^c^	25.7 (14.8, 38.3)
Blood sampling period (gestational days)^b^	231.2 ± 25.2

BMI, body mass index; DHA, docosahexaenoic acid; EPA, eicosapentaenoic acid; IQR, inter-quartile range; PFOS, perfluorooctanesulfonate; PFOA, perfluorooctanoate; SD, standard deviation.

^a^Mean ± SD.

^b^n (%).

^c^Median (IQR).

### Genotype frequencies

Table 2 shows maternal genotype frequencies. Minor homozygote frequencies were 0.6% for *PPARA* (rs1800234) CC, 0.2% for *PPARA* (rs135561) AA, 2.6% for *PPARG* (rs3856806) TT, 4.6% for *PPARGC1A* (rs2970847) TT, 22.6% for *PPARGC1A* (rs8192678) AA, 2.6% for *PPARD* (rs1053049) CC, 2.4% for *PPARD* (rs2267668) GG, 18.8% for *CAR* (rs2307424) CC, 15.1% for *CAR* (rs2501873) GG, 11.9% for *LXRA* (rs2278238) TT, 4.8% for *LXRB* (rs1405655) CC, 3.6% for *LXRB* (rs2303044) AA, and 2.8% for *LXRB* (rs4802703) AA. The genotypes of *PPARA* (rs1800234), *PPARA* (rs135561), *PPARG* (rs3856806), *PPARGC1A* (rs2970847), *PPARGC1A* (rs8192678), *PPARD* (rs1053049), *PPARD* (rs2267668), *CAR* (rs2307424), *CAR* (rs2501873), *LXRA* (rs2278238), *LXRB* (rs1405655), *LXRB* (rs2303044), and *LXRB* (rs4802703) conformed to Hardy–Weinberg equilibrium (all of *P* > 0.05). PFOS, PFOA, TG, or FA levels were not associated with the genotypes of *PPARA*, *PPARG*, *PPARGC1A*, *PPARD*, *CAR*, *LXRA*, and *LXRB* genotypes (table not shown).

**Table 2:** Maternal genotype frequencies (n = 504).

Gene name/genotype	n (%)	HWE	Gene name/genotype	n (%)	HWE
*PPARA* (T>C; rs1800234)			*PPARA* (G>A; rs135561)		
TT	443 (87.9)	χ^2^ = 1.762	GG	436 (86.5)	χ^2^ = 0.372
TC	48 (9.5)	*P* = 0.184	GA	57 (11.3)	*P* = 0.542
CC	3 (0.6)		AA	1 (0.2)	
*PPARG* (C>T; rs3856806)					
GG	358 (71.0)	χ^2^ = 0.384			
GA	123 (24.4)	*P* = 0.535			
AA	13 (2.5)				
*PPARGC1A* (C>T; rs2970847)			*PPARGC1A* (G>A; rs8192678)		
CC	301 (59.7)	χ^2^ = 0.026	GG	138 (27.4)	χ^2^ = 0.159
CT	170 (33.7)	*P* = 0.872	GA	242 (48.0)	*P* = 0.690
TT	23 (4.6)		AA	114 (22.6)	
*PPARD* (T>C; rs1053049)			*PPARD* (A>G; rs2267668)		
TT	310 (61.5)	χ^2^ = 3.502	AA	329 (65.3)	χ^2^ = 1.390
TC	171 (33.9)	*P* = 0.061	AG	153 (30.4)	*P* = 0.238
CC	13 (2.6)		GG	12 (2.4)	
*CAR* (T>C; rs2307424)			*CAR* (A>G; rs2501873)		
TT	160 (31.7)	χ^2^ = 0.876	AA	160 (31.7)	χ^2^ = 2.826
TC	268 (53.2)	*P* = 0.349	AG	258 (51.2)	*P* = 0.093
CC	95 (18.8)		GG	76 (15.1)	
*LXRA* (C>T; rs2278238)			*LXRB* (T>C; rs1405655)		
CC	207 (41.1)	χ^2^ = 0.034	TT	322 (63.9)	χ^2^ = 1.663
CT	227 (45.0)	*P* = 0.853	TC	148 (29.4)	*P* = 0.197
TT	60 (11.9)		CC	24 (4.8)	
*LXRB* (G>A; rs2303044)			*LXRB* (G>A; rs4802703)		
GG	336 (66.7)	χ^2^ = 0.510	GG	353 (70.0)	χ^2^ = 0.392
GA	140 (27.8)	*P* = 0.475	GA	127 (25.2)	*P* = 0.531
AA	18 (3.6)		AA	14 (2.8)	

Ten mothers (2.0%) did not extract DNA and analyse genotypes due to a lack of maternal blood.

Chi-square test was employed to test whether the frequency of genotype distribution conformed to the Hardy–Weinberg equilibrium.

CAR, constitutive androstane receptor; HWE, Hardy–Weinberg equilibrium; LXRA, liver X receptor alpha; LXRB, liver X receptor beta; PPARA, peroxisome proliferator-activated receptor alpha; PPARD, peroxisome proliferator-activated receptor delta; PPARG, peroxisome proliferator-activated receptor gamma; PPARGC1A, peroxisome proliferator-activated receptor gamma co-activator 1-alpha.

### Interaction between PFOS/PFOA exposure and SNP genotypes

**Figure 1:**
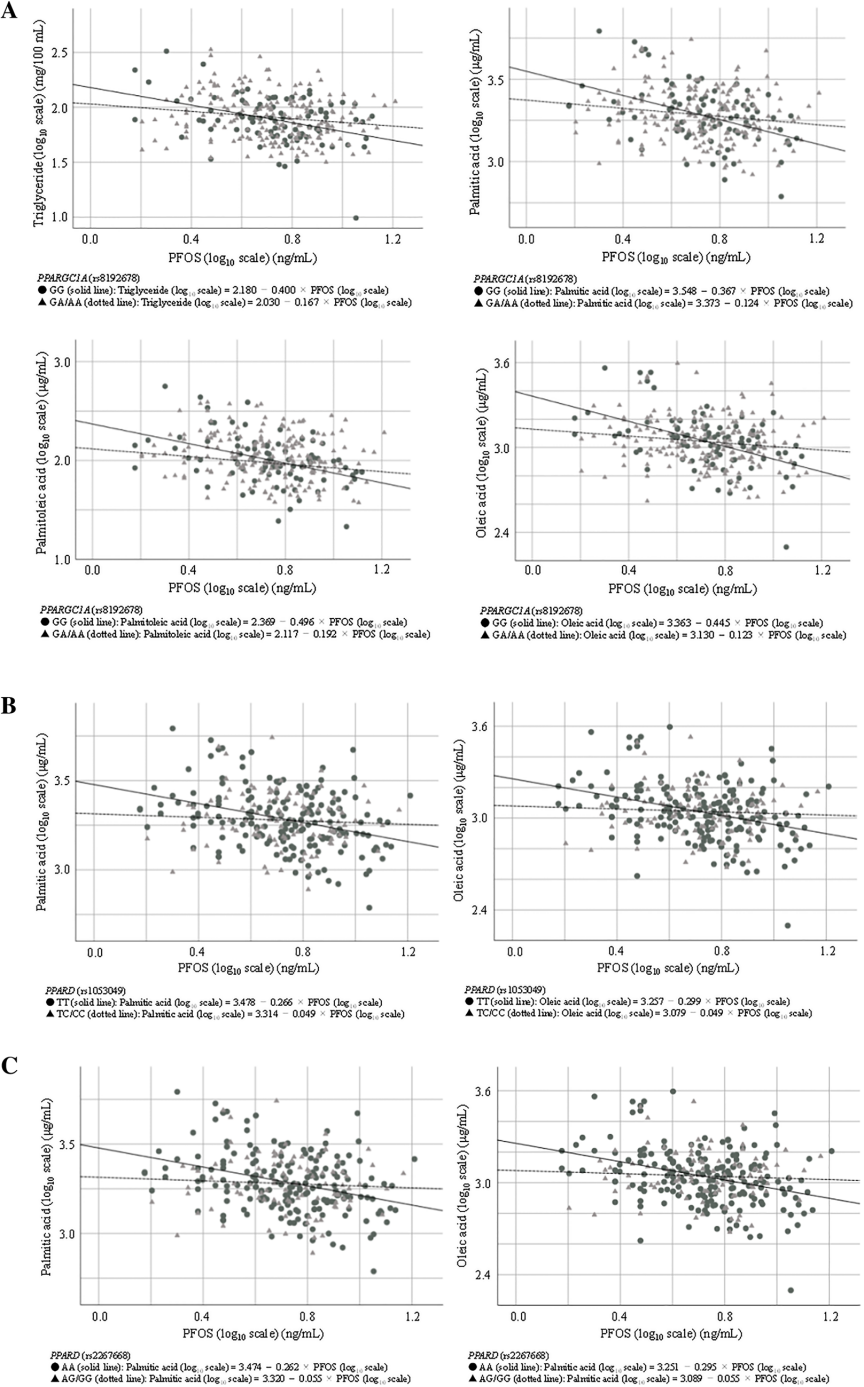
Plot of gene-environment interaction between (**A**) *PPARGC1A* (rs8192678), (**B**) *PPARD* (rs1053049), and (**C**) *PPARD* (rs2267668) and PFOS levels on fatty acid levels in serum.

Terms of interaction between PFOS exposure (log_10_ scale) and SNP genotypes were significant for one SNP reported in Table 3 and Fig. 1A–C (see also Supplementary Table 1 and Supplementary Fig. 1) for TG (log_10_ scale) (*PPARGC1A* rs8192678: *P*_*int*_ = 0.018), for three SNPs reported for palmitic acid (log_10_ scale) (*PPARGC1A* rs8192678: *P*_*int*_ = 0.004; *PPARD* rs1053049: *P*_*int*_ = 0.014; *PPARD* rs2267668: *P*_*int*_ = 0.017), for the one SNP reported for palmitoleic acid (log_10_ scale) (*PPARGC1A* rs8192678: *P*_*int*_ = 0.017), and for the three SNPs reported for oleic acid (log_10_ scale) (*PPARGC1A* rs8192678: *P*_*int*_ = 0.001; *PPARD* rs1053049: *P*_*int*_ = 0.008; *PPARD* rs2267668: *P*_*int*_ = 0.010).

**Table 3:** Association between maternal perfluorooctanesulfonate levels (log_10_ scales) and their genotypes of encoded genes in receptors on maternal triglyceride or fatty acid levels during pregnancy (log_10_ scales) (relevant only).

Gene name	Outcome	Exposure/genotype	Crude		Adjusted	
			β (95% CI)	*P* value	β (95% CI)	*P* value
*PPARGC1A* (G>A; rs8192678)	Triglyceride	PFOS	− 0.400 (− 0.606, − 0.194)	< 0.001***	− 0.389 (− 0.599, − 0.179)	< 0.001***
		*PPARGC1A* GA/AA	− 0.150 (− 0.338, 0.038)	0.115	− 0.200 (− 0.387, − 0.013)	0.037*
		PFOS × *PPARGC1A* GA/AA	0.234 (− 0.015, 0.482)	*P*_*int*_ = 0.065	0.300 (0.052, 0.548)	*P*_*int*_ = 0.018*
	Palmitic acid	PFOS	− 0.367 (− 0.527, − 0.207)	< 0.001***	− 0.372 (− 0.537, − 0.206)	< 0.001***
		*PPARGC1A* GA/AA	− 0.175 (− 0.321, − 0.029)	0.019*	− 0.204 (− 0.352, − 0.056)	0.007**
		PFOS × *PPARGC1A* GA/AA	0.243 (0.051, 0.436)	*P*_*int*_ = 0.014*	0.289 (0.093, 0.485)	*P*_*int*_ = 0.004**
	Palmitoleic acid	PFOS	− 0.496 (− 0.728, − 0.264)	< 0.001***	− 0.452 (− 0.691, − 0.213)	< 0.001***
		*PPARGC1A* GA/AA	− 0.192 (− 0.403, 0.019)	0.074	− 0.219 (− 0.432, − 0.006)	0.044*
		PFOS × *PPARGC1A* GA/AA	0.304 (0.025, 0.583)	*P*_*int*_ = 0.033*	0.345 (0.063, 0.628)	*P*_*int*_ = 0.017*
	Oleic acid	PFOS	− 0.445 (− 0.620, − 0.269)	< 0.001***	− 0.436 (− 0.615, − 0.256)	< 0.001***
		*PPARGC1A* GA/AA	− 0.233 (− 0.392, − 0.073)	0.004**	− 0.266 (− 0.426, − 0.106)	0.001**
		PFOS × *PPARGC1A* GA/AA	0.322 (0.111, 0.533)	*P*_*int*_ = 0.003**	0.377 (0.164, 0.589)	*P*_*int*_ = 0.001**
*PPARD* (T>C; rs1053049)	Palmitic acid	PFOS	− 0.266 (− 0.373, − 0.159)	< 0.001***	− 0.248 (− 0.362, − 0.134)	< 0.001***
		*PPARD* TC/CC	− 0.164 (− 0.309, − 0.018)	0.027*	− 0.186 (− 0.335, − 0.038)	0.014*
		PFOS × *PPARD* TC/CC	0.217 (0.023, 0.411)	*P*_*int*_ = 0.028*	0.250 (0.052, 0.448)	*P*_*int*_ = 0.014*
	Oleic acid	PFOS	− 0.299 (− 0.417, − 0.181)	< 0.001***	− 0.265 (− 0.389, − 0.140)	< 0.001***
		*PPARD* TC/CC	− 0.179 (− 0.339, − 0.019)	0.029*	− 0.208 (− 0.370, − 0.046)	0.012*
		PFOS × *PPARD* TC/CC	0.251 (0.038, 0.463)	*P*_*int*_ = 0.021*	0.294 (0.078, 0.510)	*P*_*int*_ = 0.008**
*PPARD* (A>G; rs2267668)	Palmitic acid	PFOS	− 0.262 (− 0.369, − 0.155)	< 0.001***	− 0.244 (− 0.357, − 0.130)	< 0.001***
		*PPARD* AG/GG	− 0.154 (− 0.300, − 0.007)	0.040*	− 0.176 (− 0.326, − 0.026)	0.021*
		PFOS × *PPARD* AG/GG	0.207 (0.012, 0.402)	*P*_*int*_ = 0.037*	0.243 (0.043, 0.443)	*P*_*int*_ = 0.017*
	Oleic acid	PFOS	− 0.295 (− 0.412, − 0.177)	< 0.001***	− 0.259 (− 0.382, − 0.136)	< 0.001***
		*PPARD* AG/GG	− 0.162 (− 0.323, − 0.001)	0.049*	− 0.190 (− 0.353, − 0.027)	0.022*
		PFOS × *PPARD* AG/GG	0.240 (0.026, 0.454)	*P*_*int*_ = 0.028*	0.286 (0.068, 0.503)	*P*_*int*_ = 0.010*

CI, confidence interval; FA, fatty acid; PFOS, perfluorooctanesulfonate; PPARD, peroxisome proliferator-activated receptor delta; PPARGC1A, peroxisome proliferator-activated receptor gamma co-activator 1-alpha.

Association between PFOS and any FA levels were tested in multiple linear regression models.

Crude: Non-adjusted.

Adjusted: Adjusted for maternal age (years; continuous), maternal smoking during the 3rd trimester (yes/no), maternal alcohol consumption during pregnancy (yes/no), annual household income (< 5/ ≥ 5 million Japanese Yen), parity (primiparous/multiparous), and sampling period (gestational days; continuous).

β (95% CI) represents change in log_10_-transformed levels of triglyceride (mg/100 mL), palmitic acid (μg/mL), palmitoleic acid (μg/mL), or oleic acid (μg/mL) for each tenfold increase in PFOS levels (ng/mL).

*P*_*int*_ represents *P* value for interaction.

*P < 0.05; **P < 0.01; ***P < 0.001.

A differential impact of PFOS exposure on reduced TG or FAs was noted between major and minor genotype groups of *PPARA* (rs1800234 and rs135561), *PPARG* (rs3856806), *LXRA* (rs2279238), and *LXRB* (rs1405655, rs2303044, and rs4802703). However, this was not indicated by gene-environment interaction between SNP genotypes and PFOS exposures (table not shown). No differential impact of PFOA exposure on reduced TG or FAs was observed between major and minor genotype groups of all 13 SNPs (table not shown). The trends of all results in the original data were similar to those of all results in the 50 pooled data with imputation (table not shown).

## Discussion

In this study, we found that the interaction between PFOS levels and *PPARGC1A* (rs8192678) and *PPARD* (rs1053049; rs2267668) genotype influences a difference in some FA levels during pregnancy. In our previous study, PFOS exposure (log_10_ scale) decreased with an observed β of − 0.168 and − 0.175 in TG, palmitic acid, palmitoleic acid, and oleic acid levels (log_10_ scale) when maternal genotypes were not considered^14^. In this study, maternal *PPARGC1A* (rs8192678), *PPARD* (rs1053049), and *PPARD* (rs2267668) genotypes were not associated with PFOS, TG, palmitic acid, palmitoleic acid, and oleic acid levels. However, when the gene-environment interaction was considered, PFOS exposure (log_10_ scale) decreased the palmitic acid, palmitoleic acid, and oleic acid levels with the observed β between − 0.452 and − 0.244; and *PPARGC1A* (rs8192678), *PPARD* (rs1053049), and *PPARD* (rs2267668) genotypes decreased the TG, palmitic acid, palmitoleic acid, and oleic acid levels with an observed β between − 0.266 and − 0.176. The results trend showed the crossover interaction due to an intercept difference. In fact, the primary effects of both environmental and genetic factors were significant, showing negative slopes in the same direction. Moreover, the magnitude of the effect of TG or FA levels for an increased PFOS level depended on *PPARGC1A* (rs8192678) or *PPARD* (rs1053049 and rs2267668) genotypes. Hence, *PPARGC1A* (rs8192678) and *PPARD* (rs1053049 and rs2267668) genotypes might facilitate the modification of FA levels by PFOS exposure during pregnancy.

First, we speculated that *PPARD* (rs1053049) TT (compared to TC/CC genotype), and *PPARD* (rs2267668) AA genotypes (compared to AG/GG genotype) decreased *PPARD* gene expression due to the PFOS-induced ligand binding of prostaglandins^37–45^. Secondly, suppression of *PPARD* activation reduced TG or FA output by decreasing glycolysis, the pentose phosphate pathway, and FA synthesis in the liver^45^. Lastly, FA levels decreased with increased PFOS exposure^14^. As PFOS is similar in structure to FAs, it can bind to apolipoproteins and disrupt lipid transport, affecting the biological properties of lipids^46^. Moreover, PFOS down-regulates a microRNA of prostaglandin-endoperoxide synthase 2^44^ and increases prostaglandins^43^. Thereafter, ligand binding of prostaglandins activates *PPARG* and *PPARD*, as previously reported^37–40,42^. *PPARD* activation upregulations the enzymes directly responsible for FA synthesis, including acetyl-CoA carboxylase β (ACCβ), fatty acid synthase (FAS), acyl-CoA thioesterase 1, and ATP citrate lyase; enzymes for elongation and modification of fatty acids including ELOVL family member 6 (ELOVL6), stearoyl-CoA desaturase 2 (SCD2), and glycerol-3-phosphate acyltransferase (GPAT); and malic enzyme in the pyruvate/malate cycle and phosphogluconate dehydrogenase (PGD) in the pentose phosphate pathway to provide reducing power for lipid synthesis^45^. The *PPARD* rs1053049 (T>C; 3′-untranslated region of exon 9) TT genotype demonstrated reduced *PPARD* gene expression levels^41^, higher levels of low-density lipoprotein cholesterol and increased risk of type 2 diabetes mellitus^47^, increased insulin sensitivity and decreased body mass with sports training or lifestyle intervention^26,48,49^. The *PPARD* rs2267668 (A>G; intron 3) AA genotype demonstrated less marked TG levels with lifestyle intervention^50^, lower dynamic balance performance^51^, higher habitual physical activity^52^, and higher peak aerobic capacity on a treadmill (VO_2 peak_)^48^. *PPARD* activation induces decreased TG levels coupled with up-regulation of genes related to lipid droplet secretion^52^. Therefore, *PPARD* (rs1053049 and rs2267668) genotypes might modify the association between PFOS levels and each FA level during pregnancy.

We speculated that *PPARGC1A* (rs8192678) GG genotype (compared to GA/AA genotype) increased both *PPARGC1A* and *PPARG* gene expression levels due to PFOS-induced ligand-binding of prostaglandins^37–40,42–44,54^; increased *PPARG* activation increased TG or FA levels via decreasing FA synthesis in the liver^45^; finally, increased PFOS exposure relatively decreased TG or FA levels^14^. *PPARGC1A* is one of the co-activators of *PPARG*, which interacts with *PPARG*. In previous studies, *PPARG* activation increased enzymes directly responsible for fatty acid synthesis including acyl-CoA synthase (ACS), fatty acid-binding protein 2 (aP2), and acyl-CoA–binding protein (ACBP)^55^. The *PPARGC1A* rs8192678 (G>A, Gly482Ser; exon 8) GG genotype demonstrated higher sports performance, athletic ability^56^, endurance performance ability^57^, hepatic adenosine triphosphate (ATP) levels^58^, *PPARGC1A* gene expression^54^, lower risk of polycystic ovarian syndrome^59^, nonalcoholic fatty acid disease^30^, type 2 diabetes mellitus^60,61^, and obesity^62^. Increased ATP levels decreased with oxidative stress induction, and acute oxidative stress decreased placental FA oxidation^63^. Other specific *PPARG* genotype modified serum lipid levels via *PPARG2* expression in adipose tissue^64^. Hence, the *PPARGC1A* (rs8192678) genotype might modify the association between PFOS levels and TG or FA levels during pregnancy.

PFOS downregulates a microRNA of the *PPARA* gene^44^, and activates *PPARA* gene expression^20,65,66^. Statins with *PPARA* activation ability interacted with *PPARA* genetic polymorphisms in the presence of *PPARGC1A* and controlled transcription of cyclic adenosine monophosphate (cAMP)-responsive element-binding protein (CREB)^67^. CREB reduces the cholesterol transporter gene *Npc1l1*, and CREB-dependent *apolipoprotein A4* (*APOA4*) activation is necessary for hepatic TG^68^. Prostaglandins downregulate *LXR* transcription^69^. *LXR* induces suppressed expression of the *apolipoprotein A5* (*APOA5*) gene, which is necessary for hepatic TG synthesis^70^. Hence, we observed the association between increased PFOS levels and decreased FA levels among specific maternal genotype (TT genotype of rs1800234 and GG genotype of rs135561 for *PPARA*; CC genotype of rs3856806 for *PPARG*; CC genotype of rs2970847 for *PPARGC1A*; AA genotype of rs2279238 for *LXRA*; TT genotype of re1405655, GG genotype of rs2303044, and GG genotype of rs4802703 for *LXRB*) (table not shown). Moreover, only the association between increased PFOS levels and decreased FA levels among specific heterozygote genotypes of maternal *CAR* were observed (TC genotype of rs2307424 and AG genotype of rs2501873) (table not shown). *CAR* activation is known to affect TG metabolism and the induction of metabolising enzymes^22,71,72^ and has been triggered by PFOS in human hepatocytes^20^. Possibly, due to limited sample size, *CAR* rs2303044 and rs2501873 were not observed to modify the association between PFOS and FA levels.

Previous studies have examined the association between prenatal PFOS, PFOA, TG, and FA levels. A Spanish study reported median PFOA levels of 2.4 ng/mL^22^, and a Norwegian study reported a value of 2.3 ng/mL^19^. Hence, our results may suggest that PFOA did not alter maternal TG or FA levels due to low levels of PFOA compared to those in previous studies^19,23^. Moreover, the percentage of smoking and alcohol consumption of women tends to be higher in Hokkaido, than in other Japanese regions^73^. The PFOS levels among smokers or alcohol consumers during pregnancy were marginally lower than those among non-smokers or non-alcohol consumers. However, our results were not affected by smoking or alcohol consumption statuses during pregnancy (table not shown). Therefore, decreased TG, palmitic acid, palmitoleic acid, or oleic acid levels for interaction between PFOS and *PPARGC1A* or *PPARD* genotype may be independent of smoking or alcohol consumption statuses during pregnancy.

The limitation of this study was that the sample size was restricted to detect gene-environment interactions; therefore, we were unable to investigate the association between PFOS/PFOA and TG/FA levels by genotype combinations. Although limited sample measurements of PFOS, PFOA, TG, and FA were performed due to anaemia during pregnancy, interactions between PFOS levels and the *PPARGC1A* or *PPARD* genotype affecting FA levels were observed in the original data. These results were similar to those of the 50 pooled data with imputation. In our results, although it was significant in the univariate test, it was not significant in the multiple comparison. Because of the small sample size, the power of statistical detection was insufficient. In the future, we would like to re-examine the association of prenatal PFOS levels and *PPARGC1A* and *PPARD* with FA levels using a group with a larger sample size. Nevertheless, the main strength of this study was that PFOS, PFOA, TG, and FA blood levels were accurately measured using column-switching LC–MS and GC–MS. Secondly, these measurements were performed during the second to third trimesters of pregnancy, as this period is indicative of rapid foetal brain growth, when maternal chemical exposure displays a critical window, and FA levels dramatically increase in the human brain^74,75^. Studying the combination of PFOS levels and polymorphisms of corresponding receptor genes revealed a decrease in blood FA levels among pregnant women in the same period. However, it is unclear whether the maternal or children’s *PPARGC1A* or *PPARD* genotype modified the association between reduced FA levels in early life and neurodevelopment in childhood and adulthood. We will attempt to determine these interactions in a further study.

In conclusion, our study demonstrates that maternal SNPs in *PPARGC1A* and *PPARD* genes modified the association between serum PFOS and TGs, or FA levels among a population of pregnant Japanese women. *PPARGC1A* and *PPARD* regulate FA metabolisms. The results of this study suggest that public health implementation of adequate FA levels for PFOS exposure during pregnancy requires minimising PFOS exposure to as low as possible and collecting information regarding high-risk genetic groups, based on informative SNPs.

## Methods

### Participants

We enrolled 514 pregnant Japanese women at 23–35 weeks of gestation who visited the Sapporo Toho Hospital in Sapporo City, Japan to participate in the “Hokkaido Study on Environment and Children’s Health (Sapporo Cohort)” between July 2002 and September 2005. The study protocol has been described previously^76^. From the time of enrolment to delivery, 10 participants dropped out due to miscarriage, stillbirth, relocation, or voluntary withdrawal. The mothers who had a miscarriage or stillbirth, who relocated, and those who voluntary withdrew from the study were not included in the study. All mothers of live-born infants were included in the study. Therefore, 504 participants were analysed in this study.

### Data collection

In the second and third trimesters, participants completed a self-administered questionnaire on smoking, alcohol consumption, annual household income, education level, and medical history. At the hospital, information regarding medical history during pregnancy was also collected.

### Maternal serum PFOS and PFOA measurements

We measured the levels of PFOS and PFOA in 447 maternal blood samples. The remaining samples were not analysed because they were not available or lacked sufficient blood volume for measurement. Of the 447 participants, 228 blood samples were acquired during pregnancy, and 159 were acquired following delivery due to patients having anemia throughout pregnancy. The 159 blood samples collected after delivery were not included in the study, and the 228 blood samples during pregnancy were included. Therefore, maternal blood sample data of 228 participants were used for examination of PFOS and PFOA during pregnancy. A 40-mL blood sample was collected from a peripheral vein following the second trimester and was used to measure maternal serum levels of PFOS and PFOA. All samples were stored at – 80 ℃ until analysis. Maternal PFOS and PFOA serum levels were measured using column-switching liquid chromatography-mass spectrometry/mass spectrometry (LC–MS/MS) at Hoshi University, Tokyo, according to a previously described protocol^77,78^. PFOS levels were detected for all participants. We assigned a 50% value (0.25 ng/mL) for 16 participants (5.9%) whose PFOS levels were below the detection limit (0.5 ng/mL).

### Maternal *PPAR*, *CAR*, and *LXR* genetic polymorphism analyses

We analysed genotypes in 494 maternal blood samples. The remaining samples were not analysed because they were not available or lacked sufficient blood volume for measurement. Therefore, we used the SNP maternal blood data of 494 participants. Maternal blood samples were collected when participants gave birth, and 400 μL of each sample was used to isolate and purify genomic DNA with a QIAamp DNA Blood Mini Kit (Qiagen GmbH, Hilden, Germany) or a Maxwell 16 DNA Purification Kit (Promega, Madison, WI, US), according to the manufacturer’s instructions^79^. We evaluated 13 SNP genotypes, namely those in *PPARA* (rs1800234 and rs135561), *PPARG* (rs3856806), *PPARGC1A* (rs2970847 and rs8192678), *PPARD* (rs1053049 and rs2267668), *CAR* (rs2307424 and rs2501873), *LXRA* (rs2279238), and *LXRB* (rs1405655, rs2303044, and rs4802703) based on analysis of high-throughput gene expression of pre-amplification (Appendix 1), real-time polymerase chain reaction (PCR) with dynamic chips (Appendix 2), and TaqMan gene-expression measurements (Appendix 3). Nine samples were randomly selected, composed of three samples, each with major homogenous, heterogeneous, and minor homogenous genotypes (with samples that were successfully genotyped using high-throughput methods). Genotyping was repeated thrice to confirm the quality for each genetic polymorphism identified using the TaqMan method. Results were 100% concordant.

### Maternal FA measurements

We measured the levels of TG and FAs in 491 maternal blood samples. The remaining samples were not analysed because they were not available or lacked sufficient blood volume for measurement. Of the 491 participants, 307 were acquired during pregnancy, and 184 were acquired following delivery due to patients having anaemia throughout pregnancy. Therefore, we used maternal blood data of 307 participants for the examination of TG and FA levels during pregnancy. FA levels in non-fasting maternal blood specimens were determined by gas chromatography-mass spectrometry (GC–MS) at Nagoya University, as described previously^18^. Nine FAs were targeted for measurement, including palmitic acid, palmitoleic acid, stearic acid, oleic acid, linoleic acid, α-linolenic acid, arachidonic acid, EPA, and DHA. The detection limits were 2.4 μg/mL for palmitic acid, 0.069 μg/mL for palmitoleic acid, 1.3 μg/mL for stearic acid, 3.6 μg/mL for oleic acid, and 2.0 μg/mL for the other FAs. Detection rates for all FAs were ≥ 99.0%, except for EPA (detection limit: 97.8%). Non-fasting blood TG levels were measured using TG E-Test Wako Kits (Wako, Osaka, Japan), following lipid extraction according to the methods described by Folch et al.^80^.

### Statistical methods

We prepared and analysed both the original dataset, as well as the 50 datasets with imputation. Regarding the 50 datasets with imputation, we imputed the missing exposures, outcomes, and confounders on maternal age (n_missing_ = 0 (0.0%)), parity (n_missing_ = 0 (0.0%)), maternal smoking in the third trimesters (n_missing_ = 0 (0.0%)), maternal alcohol consumption during pregnancy (n_missing_ = 0 (0.0%)), annual household income (n_missing_ = 16 (3.2%)), blood sampling period (n_missing_ = 191 (37.9%)), PFOS/PFOA (n_missing_ = 216 (42.9%)), genotypes of 13 SNPs (n_missing_ = 10 (2.0%)), and TG/FAs (n_missing_ = 197 (39.1%)) using the multiple imputation package for SPSS version 26 (IBM Corp. Armonk, NY, USA). First, we analysed the characteristics and all PFOS, PFOA, TG, and FA levels in study participants. Chi-square test was employed to test whether the frequency of genotype distribution conformed to the Hardy–Weinberg equilibrium. Second, due to the skewed distributions, we treated the levels of PFOS, PFOA, TG, and FAs as variables on a log_10_ scale or four quartiles. Formulae in multiple linear regression models and least squares means (LSMs) among all participants and the participants with each genotype are defined as log_10_-transformed TG or each FA level = intercept + estimate (β)_1_ (PFOS or PFOA levels (log_10_ scale or four quartiles)) + β_2_ (predicted low-risk genotype = 0/high-risk genotype = 1) (among all participants only) + β_3_ ((log_10_ scale of PFOS or PFOA levels) × (predicted low-risk genotype = 0/high-risk genotype = 1)) (among all participants only) + β_4_ (maternal age (years; continuous)) (adjusted) + β_5_ (no = 0/yes = 1 of maternal smoking in the 3^rd^ trimester) (adjusted) + β_6_ (no = 0/yes = 1 of maternal alcohol consumption during pregnancy) (adjusted) + β_7_ (< 5 = 0/ ≥ 5 = 1 (million Japanese Yen) of annual household income) (adjusted) + β_8_ (primiparous = 0/multiparous = 1 of parity) (adjusted) + β_9_ (blood sampling periods; (gestational days; continuous)) (adjusted). Moreover, LSMs and the 95% confidence interval (CI) were calculated, and LSMs and the CI were back transformed from log_10_ scale to normal values. Data were considered statistically significant at *P* < 0.05. All statistical analyses were performed using SPSS version 26 (IBM Corp.), except for LSMs, which were analysed using JMP Pro 14 (SAS Institute Inc., Cary, NC, USA).

### Ethics

Written informed consent was obtained from all participants. All experimental protocols were approved by the Institutional Ethical Board for Human Genome and Genome Studies at the Hokkaido University Graduate School of Medicine and the Hokkaido University Center for Environmental and Health Sciences (registration number: 119; registration date: 5th Sep 2019). All methods were carried out in accordance with relevant guidelines and regulations, specifically: the Declaration of Helsinki (World Medical Association), the Ethical Guidelines for Epidemiological Research (Ministry of Education, Culture, Sports, Science and Technology, and Ministry of Health, Labour and Welfare, Japan), the Ethical Guidelines for Human Genome/Gene Analysis Research (Ministry of Education, Culture, Sports, Science and Technology, Ministry of Health, Labour and Welfare, and Ministry of Economy, Trade and Industry, Japan), the Ethical Guidelines for Medical and Health Research Involving Human Subjects (Ministry of Education, Culture, Sports, Science and Technology, and Ministry of Health, Labour and Welfare, Japan), the Guidelines of the Council for International Organization of Medical Sciences (World Health Organization), and the Strengthening the Reporting of Observational Studies in Epidemiology (STROBE) Statement: Guidelines for Reporting Observational Studies (International collaborative initiative of epidemiologists, methodologists, statisticians, researchers and journal editors involved in the conduct and dissemination of observational studies).

## Supplementary Information

Supplementary Information.

## Data Availability

The data and materials used to derive our conclusions are unsuitable for public deposition due to ethical restrictions and specific legal framework in Japan. It is prohibited by the Act on the Protection of Personal Information (Act No. 57 of May 30, 2003, amended on September 9, 2015) to publicly deposit data containing personal information. The Ethical Guidelines for Epidemiological Research enforced by the Japan Ministry of Education, Culture, Sports, Science and Technology and the Ministry of Health, Labour and Welfare also restrict the open sharing of the epidemiologic data. All inquiries about access to data should be sent to rkishi@med.hokudai.ac.jp. The person responsible for handling inquiries sent to this e-mail address is Professor Reiko Kishi, Principal Investigator of the Hokkaido Study on Environment and Children's Health, Center for Environmental and Health Sciences, Hokkaido University.

## References

[1] Wang Z (2020). Role of rare and low-frequency variants in gene-alcohol interactions on plasma lipid levels. Circ. Genom. Precis. Med..

[2] Kwon EJ (2016). Prenatal exposure to perfluorinated compounds affects birth weight through GSTM1 polymorphism. J. Occup. Environ. Med..

[3] Wen HJ, Wang SL, Chen PC, Guo YL (2019). Prenatal perfluorooctanoic acid exposure and glutathione s-transferase T1/M1 genotypes and their association with atopic dermatitis at 2 years of age. PLoS ONE.

[4] Yang W, Mao S, Qu B, Zhang F, Xu Z (2017). Association of peroxisome proliferator-activated receptor delta and additional gene-smoking interaction on cardiovascular disease. Clin. Exp. Hypertens..

[5] Ding X (2016). Interaction between peroxisome proliferator-activated receptor gamma and smoking on cardiovascular disease. Physiol. Behav..

[6] Kobayashi S (2016). Combined effects of AHR, CYP1A1, and XRCC1 genotypes and prenatal maternal smoking on infant birth size: Biomarker assessment in the Hokkaido study. Reprod. Toxicol..

[7] Kobayashi S (2017). Dioxin-metabolizing genes in relation to effects of prenatal dioxin levels and reduced birth size: The Hokkaido study. Reprod. Toxicol..

[8] Kobayashi S (2017). Modification of adverse health effects of maternal active and passive smoking by genetic susceptibility: Dose-dependent association of plasma cotinine with infant birth size among Japanese women-the Hokkaido study. Reprod. Toxicol..

[9] Washino N (2009). Correlations between prenatal exposure to perfluorinated chemicals and reduced fetal growth. Environ. Health Perspect..

[10] Kobayashi S (2017). Effects of prenatal perfluoroalkyl acid exposure on cord blood IGF2/H19 methylation and ponderal index: The Hokkaido study. J. Expo. Sci. Environ. Epidemiol..

[11] Okada E (2014). Prenatal exposure to perfluoroalkyl acids and allergic diseases in early childhood. Environ. Int..

[12] Goudarzi H (2016). Effects of prenatal exposure to perfluoroalkyl acids on prevalence ofallergic diseases among 4-year-old children. Environ. Int..

[13] Goudarzi H (2017). Prenatal exposure to perfluoroalkyl acids and prevalence of infectious diseases up to 4years of age. Environ. Int..

[14] Kishi R (2015). The association of prenatal exposure to perfluorinated chemicals with maternal essential and long-chain polyunsaturated fatty acids during pregnancy and the birth weight of their offspring: The Hokkaido study. Environ. Health Perspect..

[15] Matilla-Santander N (2017). Exposure to perfluoroalkyl substances and metabolic outcomes in pregnant women: Evidence from the Spanish INMA birth cohorts. Environ. Health Perspect..

[16] Maisonet M (2015). Prenatal exposure to perfluoroalkyl acids and serum testosterone concentrations at 15 years of age in female ALSPAC study participants. Environ. Health Perspect..

[17] Halldorsson TI (2012). Prenatal exposure to perfluorooctanoate and risk of overweight at 20 years of age: A prospective cohort study. Environ. Health Perspect..

[18] Jia X (2015). Association of maternal whole blood fatty acid status during the prenatal period with term birth dimensions: A cross-sectional study. J. Perinat. Med..

[19] Starling AP (2014). Perfluoroalkyl substances and lipid concentrations in plasma during pregnancy among women in the Norwegian mother and child cohort study. Environ. Int..

[20] Bjork JA, Butenhoff JL, Wallace KB (2011). Multiplicity of nuclear receptor activation by PFOA and PFOS in primary human and rodent hepatocytes. Toxicology.

[21] Edwards PA, Kennedy MA, Mak PA (2002). LXRs; oxysterol-activated nuclear receptors that regulate genes controlling lipid homeostasis. Vascul. Pharmacol..

[22] Maglich JM, Lobe DC, Moore JT (2009). The nuclear receptor CAR (NR1I3) regulates serum triglyceride levels under conditions of metabolic stress. J. Lipid Res..

[23] Popeijus HE (2014). Fatty acid chain length and saturation influences PPARα transcriptional activation and repression in HepG2 cells. Mol. Nutr. Food Res..

[24] Naito H (2007). Differential effects of aging, drinking and exercise on serum cholesterol levels dependent on the PPARA-V227A polymorphism. J. Occup. Health..

[25] Cresci S (2010). A PPARα promoter variant impairs ERR-dependent transactivation and decreases mortality after acute coronary ischemia in patients with diabetes. PLoS ONE.

[26] Leońska-Duniec A (2018). The polymorphisms of the PPARD gene modify post-training body mass and biochemical parameter changes in women. PLoS ONE.

[27] Lin J (2019). PPARG rs3856806 C>T polymorphism increased the risk of colorectal cancer: A case-control study in eastern Chinese Han population. Front Oncol..

[28] Vimaleswaran KS (2006). Effect of polymorphisms in the PPARGC1A gene on body fat in Asian Indians. Int. J. Obes..

[29] Lin YC (2013). A common variant in the peroxisome proliferator-activated receptor-γ coactivator-1α gene is associated with nonalcoholic fatty liver disease in obese children. Am. J. Clin. Nutr..

[30] Kaupert LC (2013). The effect of fetal androgen metabolism-related gene variants on external genitalia virilization in congenital adrenal hyperplasia. Clin. Genet..

[31] Lima LO (2013). PPARA, RXRA, NR1I2 and NR1I3 gene polymorphisms and lipid and lipoprotein levels in a southern Brazilian population. Mol. Biol. Rep..

[32] Agarwal S (2006). Liver X receptor-α polymorphisms (rs11039155 and rs2279238) are associated with susceptibility to vitiligo. Meta Gene..

[33] Wang Z (2016). Nuclear receptor NR1H3 in familial multiple sclerosis. Neuron.

[34] Andersen V (2011). Polymorphisms in NF-κB, PXR, LXR, PPARγ and risk of inflammatory bowel disease. World J. Gastroenterol..

[35] Han M (2014). Liver X receptor gene polymorphisms in tuberculosis: Effect on susceptibility. PLoS ONE.

[36] Solaas K (2010). Suggestive evidence of associations between liver X receptor β polymorphisms with type 2 diabetes mellitus and obesity in three cohort studies: HUNT2 (Norway), MONICA (France) and HELENA (Europe). BMC Med. Genet..

[37] Ding Y (2012). Gene-gene interaction between PPARδ and PPARγ is associated with abdominal obesity in a Chinese population. J. Genet. Genomics..

[38] Ding X (2020). The Impact of PPARD and PPARG polymorphisms on glioma risk and prognosis. Sci. Rep..

[39] Gillio-Meina C (2009). Expression patterns and role of prostaglandin-endoperoxide synthases, prostaglandin E synthases, prostacyclin synthase, prostacyclin receptor, peroxisome proliferator-activated receptor delta and retinoid x receptor alpha in rat endometrium during artificially-induced decidualization. Reproduction.

[40] Kliewer SA (2001). Peroxisome proliferator-activated receptors: From genes to physiology. Recent Prog. Horm. Res..

[41] Ordelheide AM (2011). In vitro responsiveness of human muscle cell peroxisome proliferator-activated receptor δ reflects donors' insulin sensitivity in vivo. Eur. J. Clin. Investig..

[42] San-Segundo L (2016). Alterations in gene expression levels provide early indicators of chemical stress during *Xenopus laevis* embryo development: A case study with perfluorooctane sulfonate (PFOS). Ecotoxicol. Environ. Saf..

[43] Wang X (2004). Prostaglandin E_2_ is a product of induced prostaglandin-endoperoxide synthase 2 and microsomal-type prostaglandin E synthase at the implantation site of the hamster. J. Biol. Chem..

[44] Xu Y (2020). Association between serum concentrations of perfluoroalkyl substances (PFAS) and expression of serum microRNAs in a cohort highly exposed to PFAS from drinking water. Environ. Int..

[45] Lee CH (2006). PPARdelta regulates glucose metabolism and insulin sensitivity. Proc. Natl. Acad. Sci. USA..

[46] Chen YM, Guo LH (2009). Fluorescence study on site-specific binding of perfluoroalkyl acids to human serum albumin. Arch. Toxicol..

[47] Vänttinen M (2005). Single nucleotide polymorphisms in the peroxisome proliferator-activated receptor delta gene are associated with skeletal muscle glucose uptake. Diabetes.

[48] Stefan N (2007). Genetic variations in PPARD and PPARGC1A determine mitochondrial function and change in aerobic physical fitness and insulin sensitivity during lifestyle intervention. J. Clin. Endocrinol. Metab..

[49] Thamer C (2008). Variations in PPARD determine the change in body composition during lifestyle intervention: A whole-body magnetic resonance study. J. Clin. Endocrinol. Metab..

[50] Nishida Y (2018). Influence of single-nucleotide polymorphisms in PPAR-δ, PPAR-γ, and PRKAA2 on the changes in anthropometric indices and blood measurements through exercise-centered lifestyle intervention in Japanese middle-aged men. Int. J. Mol. Sci..

[51] Cao Y (2019). Polymorphism of the PPARD gene and dynamic balance performance in Han Chinese children. Hereditas.

[52] Gielen M (2014). Heritability and genetic etiology of habitual physical activity: A twin study with objective measures. Genes Nutr..

[53] Shi HB (2018). Peroxisome proliferator-activated receptor delta regulates lipid droplet formation and transport in goat mammary epithelial cells. J. Dairy Sci..

[54] Mirzaei K (2012). An exonic peroxisome proliferator-activated receptor-γ coactivator-1α variation may mediate the resting energy expenditure through a potential regulatory role on important gene expression in this pathway. J. Nutrigenet. Nutrigenomics..

[55] Ahmadian M (2013). PPARγ signaling and metabolism: The good, the bad and the future. Nat. Med..

[56] Tharabenjasin P, Pabalan N, Jarjanazi H (2019). Association of PPARGC1A Gly428Ser (rs8192678) polymorphism with potential for athletic ability and sports performance: A meta-analysis. PLoS ONE.

[57] Eynon N (2010). Do PPARGC1A and PPARalpha polymorphisms influence sprint or endurance phenotypes?. Scand. J. Med. Sci. Sports..

[58] Gancheva S (2016). Variants in genes controlling oxidative metabolism contribute to lower hepatic ATP independent of liver fat content in type 1 diabetes. Diabetes.

[59] Reddy TV (2018). Polymorphisms in the TFAM and PGC1-α genes and their association with polycystic ovary syndrome among south Indian women. Gene.

[60] Sharma R (2018). Association of PGC-1α gene with type 2 diabetes in three unrelated endogamous groups of north-west India (Punjab): A case-control and meta-analysis study. Mol. Genet. Genomics..

[61] Xia W (2019). Systematic meta-analysis revealed an association of PGC-1α rs8192678 polymorphism in type 2 diabetes mellitus. Dis. Markers..

[62] Zamaninour N (2018). Peroxisome proliferator-activated receptor gamma coactivator 1α variation: A closer look at obesity onset age and its related metabolic status and body composition. Appl. Physiol. Nutr. Metab..

[63] Thomas MM (2019). Oxidative stress impairs fatty acid oxidation and mitochondrial function in the term placenta. Reprod. Sci..

[64] Deeb SS (1998). A Pro12Ala substitution in PPARgamma2 associated with decreased receptor activity, lower body mass index and improved insulin sensitivity. Nat. Genet..

[65] Wolf CJ (2008). Activation of mouse and human peroxisome proliferator-activated receptor alpha by perfluoroalkyl acids of different functional groups and chain lengths. Toxicol. Sci..

[66] Shipley JM (2004). trans-activation of PPARalpha and induction of PPARalpha target genes by perfluorooctane-based chemicals. Toxicol. Sci..

[67] Roy A (2016). Identification and characterization of PPARα ligands in the hippocampus. Nat. Chem. Biol..

[68] Nakagawa Y, Shimano H (2018). CREBH regulates systemic glucose and lipid metabolism. Int. J. Mol. Sci..

[69] Alba G (2012). Transcription of liver X receptor is down-regulated by 15-deoxy-Δ(12,14)-prostaglandin J(2) through oxidative stress in human neutrophils. PLoS ONE.

[70] Jakel H (2004). The liver X receptor ligand T0901317 down-regulates APOA5 gene expression through activation of SREBP-1c. J. Biol. Chem..

[71] Ferguson SS (2002). Regulation of human CYP2C9 by the constitutive androstane receptor: Discovery of a new distal binding site. Mol. Pharmacol..

[72] Roth A (2008). Sterol regulatory element binding protein 1 interacts with pregnane X receptor and constitutive androstane receptor and represses their target genes. Pharmacogenet. Genomics..

[73] Matsuzaki M (2014). Effects of lifestyle factors on urinary oxidative stress and serum antioxidant markers in pregnant Japanese women: A cohort study. Biosci. Trends..

[74] Clandinin MT (1980). Intrauterine fatty acid accretion rates in human brain: Implications for fatty acid requirements. Early. Hum. Dev..

[75] Selevan SG, Kimmel CA, Mendola P (2000). Identifying critical windows of exposure for children's health. Environ. Health Perspect..

[76] Kishi R (2017). The Hokkaido birth cohort study on environment and children's health: Cohort profile-updated 2017. Environ. Health Prev. Med..

[77] Nakata H (2005). Development of an analytical method for perfluorochemicals in human plasma and blood by liquid chromatography-tandem mass spectrometry coupled with solid-phase extraction using a column-switching technique. Organohalogen Compd..

[78] Inoue K (2004). Determination of perfluorooctane sulfonate, perfluorooctanoate and perfluorooctane sulfonylamide in human plasma by column-switching liquid chromatography-electrospray mass spectrometry coupled with solid-phase extraction. J. Chromatogr. B Analyt. Technol. Biomed. Life Sci..

[79] Kobayashi S (2013). Genetic association of aromatic hydrocarbon receptor (AHR) and cytochrome P450, family 1, subfamily A, polypeptide 1 (CYP1A1) polymorphisms with dioxin blood concentrations among pregnant Japanese women. Toxicol. Lett..

[80] Folch J, Lees M, Shoane Stanley GH (1957). A simple method for the isolation and purification of total lipids from animal tissues. J. Biol. Chem..

