# Supplementary material for: Associations among perfluorooctanesulfonic/perfluorooctanoic acid levels, nuclear receptor gene polymorphisms, and lipid levels in pregnant women in the Hokkaido study
Source: Sci Rep. 2021 May 11;11:9994. doi: 10.1038/s41598-021-89285-2 (PMC8113244; doi:10.1038/s41598-021-89285-2)
Supplement: Supplementary file 1 — Supplementary Information. [file 41598_2021_89285_MOESM1_ESM.pdf]

Supplemental materials

Title: Associations among perfluorooctanesulfonic/perfluorooctanoic acid levels, nuclear receptor gene polymorphisms, and lipid levels in pregnant women in the Hokkaido study

Authors: Sumitaka Kobayashi, Fumihiro Sata, Houman Goudarzi, Atsuko Araki, Chihiro Miyashita, Seiko Sasaki, Emiko Okada, Yusuke Iwasaki, Tamie Nakajima, Reiko Kishi

Appendix 1. High-throughput pre-amplification

Appendix 2. High-throughput real-time PCR on dynamic chips

Appendix 3. Real-time PCR with TaqMan probes

Supplementary Table S1. Association of maternal perfluorooctanesulfonate levels (log_{10} scales) with maternal triglyceride or fatty acid levels during pregnancy (log_{10} scales), stratified by maternal *PPARGC1A* (rs8192678) and *PPARD* (rs1053049 and rs2267668) genotypes

Appendix 1. High-throughput pre-amplification

To enrich the amount of template, each test sample was pre-amplified using Qiagen 2× Multiplex PCR Master Mix (Qiagen GmbH) according to the manufacturer's protocol. We performed the assays in 96-well format. Mixtures for each sample (final volume 5.0 μL) contained 2.5 μL Qiagen 2× Multiplex PCR Master Mix, 0.5 μL 10× SNP type Specific Target Amplification (STA) Primer Pool (from a mixture containing 96 μL 100 μM SNPtype Assay STA primer, 96 μL 100 μM SNPtype assay LSP, and 208.0 μL DNA suspension Buffer [TEKnova, Hollister, CA, US]), 1.25 μL of genomic DNA, and water added to a total volume of 5.0 μL. Pre-amplifications were carried out on a Verity 96-Well Thermal Cycler (Applied Biosystems, Foster, CA, US) using the following protocol from the manufacturer's instructions: initial denaturation at 95 °C for 15 min, followed by 14 cycles of 15 s at 95 °C and 4 min at 60 °C. Each pre-amplified product was diluted 100-fold prior to its use as a template in subsequent PCR reactions.

Appendix 2. High-throughput real-time PCR on dynamic chips

Fluidigm 96.96 real-time PCR runs were performed according to the manufacturer's instructions (Fluidigm Corp., South San Francisco, CA, US). Prior to performing real-time PCR, sample and assay mixtures were prepared individually. Each sample mixture (final volume: 6.0 μL) contained 3.0 μL Biotium 2× Fast Probe Master Mix (Biotium Inc., Fremont, CA, US), 0.3 μL SNPtype 20× sample Loading Reagent (Fluidigm Corp.), 0.1 μL 60× SNPtype Reagent (Fluidigm Corp.), 0.036 μL 50× ROX (Invitrogen, Waltham, MA, US), 0.064 μL water, and 2.5μL diluted pre-amplification product as the template. Each assay mixture (final volume 5.0 μL) contained 2.5 μL 2× Assay Loading Reagent (Fluidigm Corp.), 1.5 μL water, and 1.0 μL SNPtype Assay Mix (from a mixture containing 3.0 μL SNPtype Assay ASP1/ASP2 [Fluidigm Corp.], 8.0 μL SNPtype Assay LSP [Fluidigm Corp.], and 29.0 μL DNA suspension buffer [TEKnova]). Sample and assay mixtures were loaded into separate reaction chambers on the 96.96 Dynamic Array IFC chip (Fluidigm Corp.) in an IFC Controller HX instrument (Fluidigm Corp.), following the addition of 5 μL assay mixture and 6 μL sample mixture to the assay inlet or sample inlet. The following thermal cycling protocol ran using an FC1 cycler (Fluidigm Corp.): 95 °C initial denaturation for 5 min; one cycle of 15 s at 95 °C, 45 s at 64 °C, and 15 s at 72 °C; one cycle of 15 s at 95 °C, 45 s at 63 °C, and 15 s at 72 °C; one cycle of 15 s at 95 °C, 45 s at 62 °C, and 15 s at 72 °C; one cycle of 15 s at 95 °C, 45 s at 61 °C, and 15 s at 72 °C; and 34 cycles of 15 s at 95 °C, 45 s at 60 °C, and 15 s at 72 °C. Fluorescence was measured using an EP1 reader (Fluidigm Corp.) equipped with SNP Genotyping Analysis Software version 3.0.2. (Fluidigm Corp.). We used only maternal genotypes that were tested successfully in duplicate by the high-throughput method.

Appendix 3. Real-time PCR with TaqMan probes

Maternal samples that failed the high-throughput SNP analysis were re-evaluated using TaqMan PCR probes. Thirteen SNP genotypes were tested using a StepOne Real-Time PCR System (Applied Biosystems) and the fluorogenic 5'-nuclease assay with TaqMan Minor Groove Binder probes (Applied Biosystems) according to the manufacturer's protocols. Each reaction contained 2 ng/μL genomic DNA, TaqMan Assay-On Demand SNP Genotyping Assay Mix (Applied Biosystems), TaqMan GTXpress Master Mix (Applied Biosystems), and No AmpErase UNG (Applied Biosystems) in a total volume of 10 μL. The reaction conditions were as follows: 20 seconds at 95.0°C, followed by 40 cycles of 3 seconds at 95°C and 20 seconds at 60°C. Allelic discrimination was determined by measuring the fluorescence of two dyes at 60°C. We used only the maternal genotypes that were tested successfully in duplicate with TaqMan probes.

Supplementary Table S1. Association of maternal perfluorooctanesulfonate levels (log_{10} scales) with maternal triglyceride or fatty acid levels during pregnancy (log_{10} scales), stratified by maternal *PPARGC1A* (rs8192678) and *PPARD* (rs1053049 and rs2267668) genotypes

		Exposure: PFOS			
Outcome	Gene name/ genotype	Crude β (95% CI)	*P* value	Adjusted β (95% CI)	*P* value
	***PPARGC1A* (G>A; rs8192678)**				
Triglyceride	GG (n = 138)	-0.400 (-0.609, -0.191)	<0.001***	-0.449 (-0.677, -0.221)	<0.001***
	GA/AA (n = 356)	-0.167 (-0.305, -0.028)	0.018*	-0.060 (-0.209, 0.088)	0.424
Palmitic acid	GG (n = 138)	-0.367 (-0.533, -0.201)	<0.001***	-0.399 (-0.582, -0.216)	<0.001***
	GA/AA (n = 356)	-0.124 (-0.230, -0.017)	0.077	-0.131 (-0.372, 0.111)	0.283
Palmitoleic acid	GG (n = 138)	-0.496 (-0.734, -0.258)	<0.001***	-0.481 (-0.741, -0.220)	<0.001***
	GA/AA (n = 356)	-0.192 (-0.346, -0.037)	0.015*	-0.081 (-0.249, 0.087)	0.341
Oleic acid	GG (n = 138)	-0.445 (-0.626, -0.264)	<0.001***	-0.491 (-0.692, -0.291)	<0.001***
	GA/AA (n = 356)	-0.123 (-0.239, -0.006)	0.039*	-0.032 (-0.158, 0.093)	0.615
	***PPARD* (T>C; rs1053049)**				
Palmitic acid	TT (n = 310)	-0.266 (-0.375, -0.157)	<0.001***	-0.263 (-0.382, -0.145)	<0.001***
	TC/CC (n = 184)	-0.049 (-0.207, 0.109)	0.541	0.024 (-0.155, 0.202)	0.794
Oleic acid	TT (n = 310)	-0.299 (-0.423, -0.176)	<0.001***	-0.269 (-0.401, -0.137)	<0.001***
	TC/CC (n = 184)	-0.049 (-0.214, 0.116)	0.558	0.017 (-0.168, 0.203)	0.852
	***PPARD* (A>G; rs2267668)**				
Palmitic acid	AA (n = 329)	-0.262 (-0.369, -0.154)	<0.001***	-0.255 (-0.371, -0.138)	<0.001***
	AG/GG (n = 165)	-0.055 (-0.218, 0.109)	0.509	0.021 (-0.168, 0.210)	0.824
Oleic acid	AA (n = 329)	-0.295 (-0.416, -0.173)	<0.001***	-0.263 (-0.393, -0.133)	<0.001***
	AG/GG (n = 165)	-0.055 (-0.223, 0.113)	0.519	0.017 (-0.174, 0.208)	0.859

Abbreviations: CI, confidence interval; FA, fatty acid; PFOS, perfluorooctanesulfonate; PPARD, peroxisome proliferator-activated receptor delta; PPARGC1A, peroxisome proliferator-activated receptor gamma co-activator 1-alpha.
Association between PFOS and any FA levels were tested in multiple linear regression models.
Crude: Non-adjusted.
Adjusted: Adjusted for maternal age (years; continuous), maternal smoking during the 3rd trimester (yes/no), maternal alcohol consumption during pregnancy (yes/no), annual household income (< 5/≥ 5 million Japanese Yen), parity (primiparous/multiparous), and sampling period (gestational days; continuous).
β (95% CI) represents change in log_{10}-transformed levels of triglyceride (mg/100 mL), palmitic acid (μg/mL), palmitoleic acid (μg/mL), or oleic acid (μg/mL) for each 10-fold increase in PFOS levels (ng/mL).
* $P < 0.05$; ** $P < 0.01$; *** $P < 0.001$.

Supplementary Figure legends

Supplementary Fig S1. Association of maternal triglyceride or fatty acid levels with maternal perfluorooctanesulfonate levels during pregnancy (quartile levels) stratified by maternal *PPARGC1A* (rs8192678) and *PPARD* (rs1053049 and rs2267668) genotypes and association of maternal triglyceride or fatty acid levels during pregnancy with *PPARGC1A* (rs8192678) and *PPARD* (rs1053049 and rs2267668) genotypes among all

Figures show the combination with (A) triglyceride, (B) palmitic acid, (C) palmitoleic acid, or (D) oleic acid and *PPARGC1A* (rs8192678); the combination with (E) palmitic acid or (F) oleic acid and *PPARD* (rs1053049); and the combination with (G) palmitic acid or (H) oleic acid and *PPARD* (rs2267668).

Least-squares means are adjusted for maternal age (years; continuous), maternal smoking during the 3rd trimester (yes/no), maternal alcohol consumption during pregnancy (yes/no), annual household income (<5/≥5 million Japanese Yen), parity (primiparous/multiparous), and sampling period (gestational days; continuous).

PFOS quartile 1 (Q1), 1.5 to < 4.0 ng/mL; quartile 2 (Q2), 4.0 to <5.4 ng/mL; quartile 3 (Q3), 5.4 to <7.4 ng/mL; quartile 4 (Q4), 7.4 to 16.2 ng/mL.

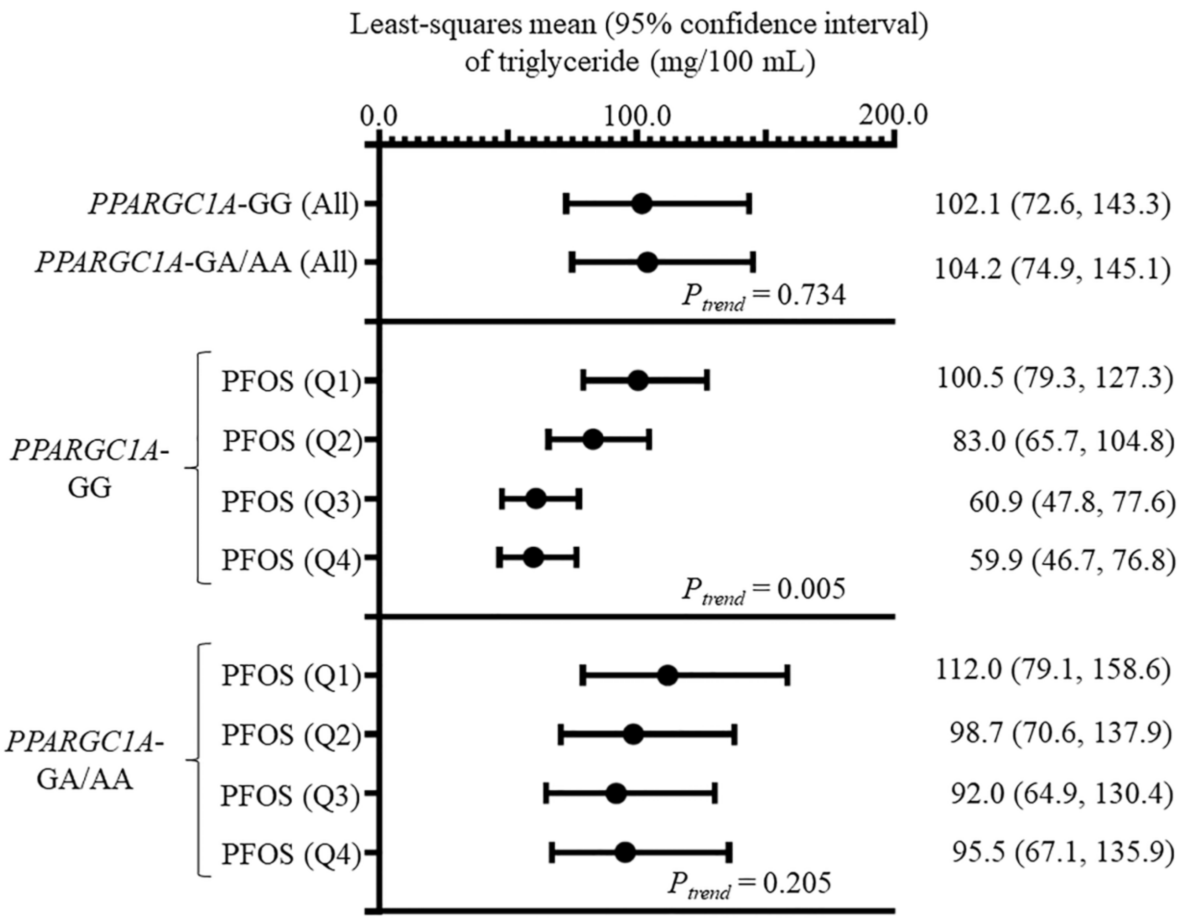

Supplementary Fig. S1 (A)

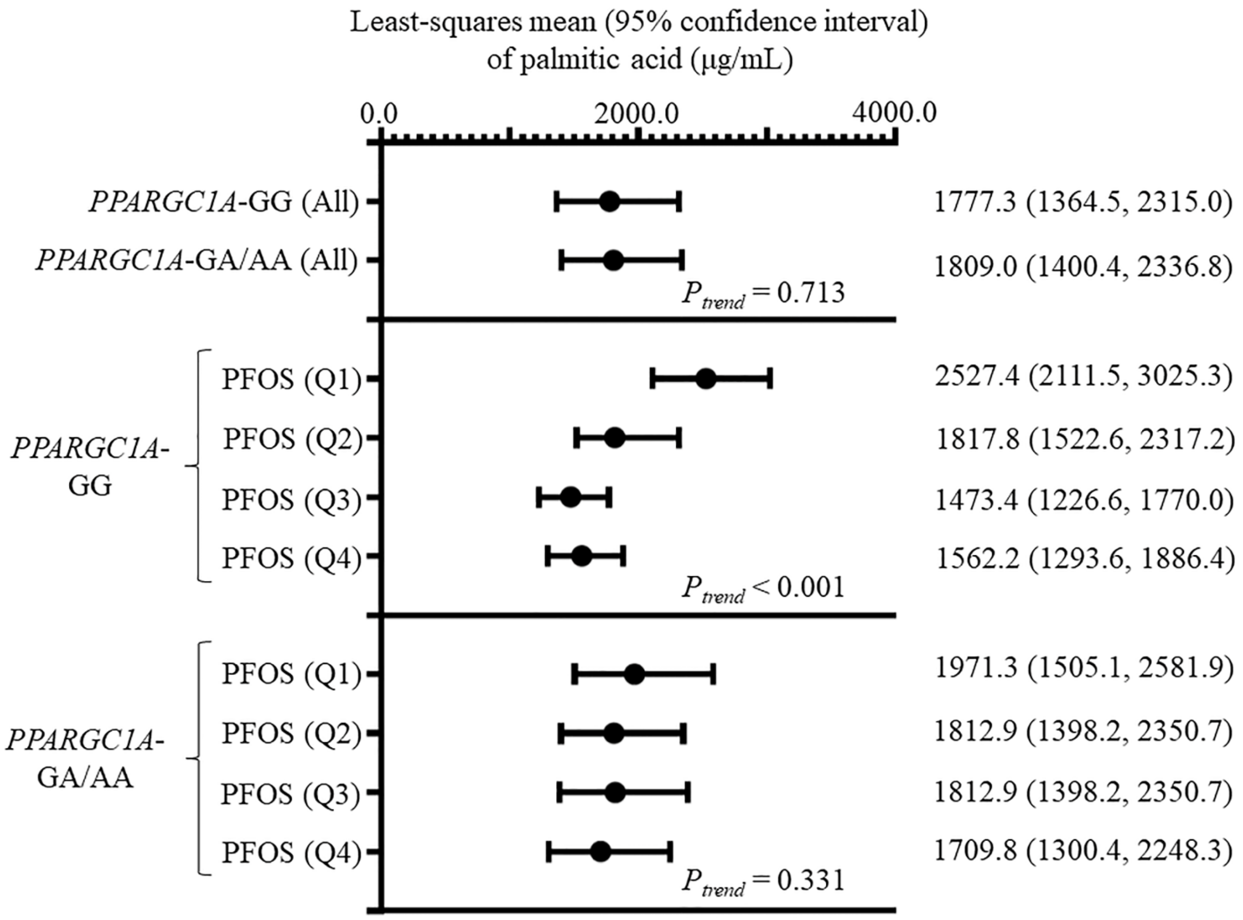

Supplementary Fig. S1 (B)

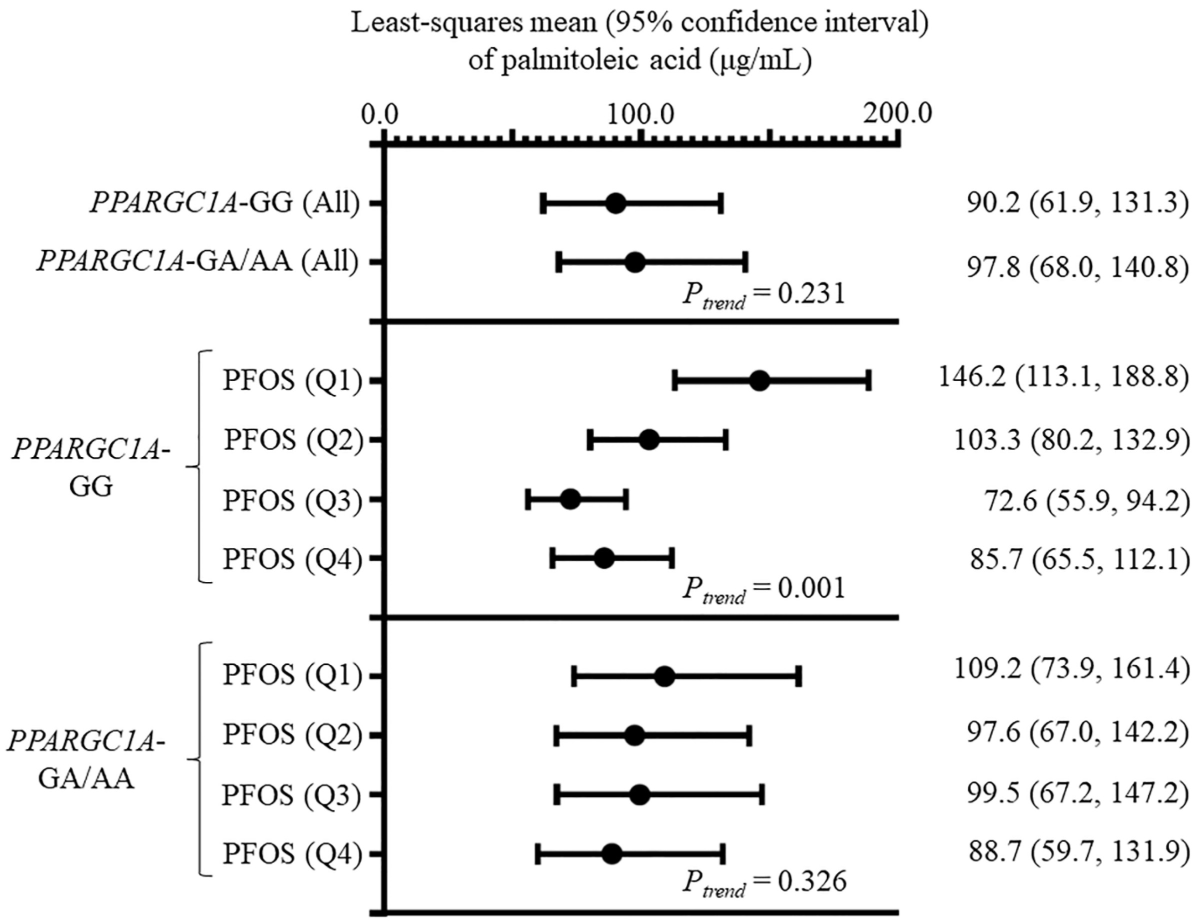

Supplementary Fig. S1 (C)

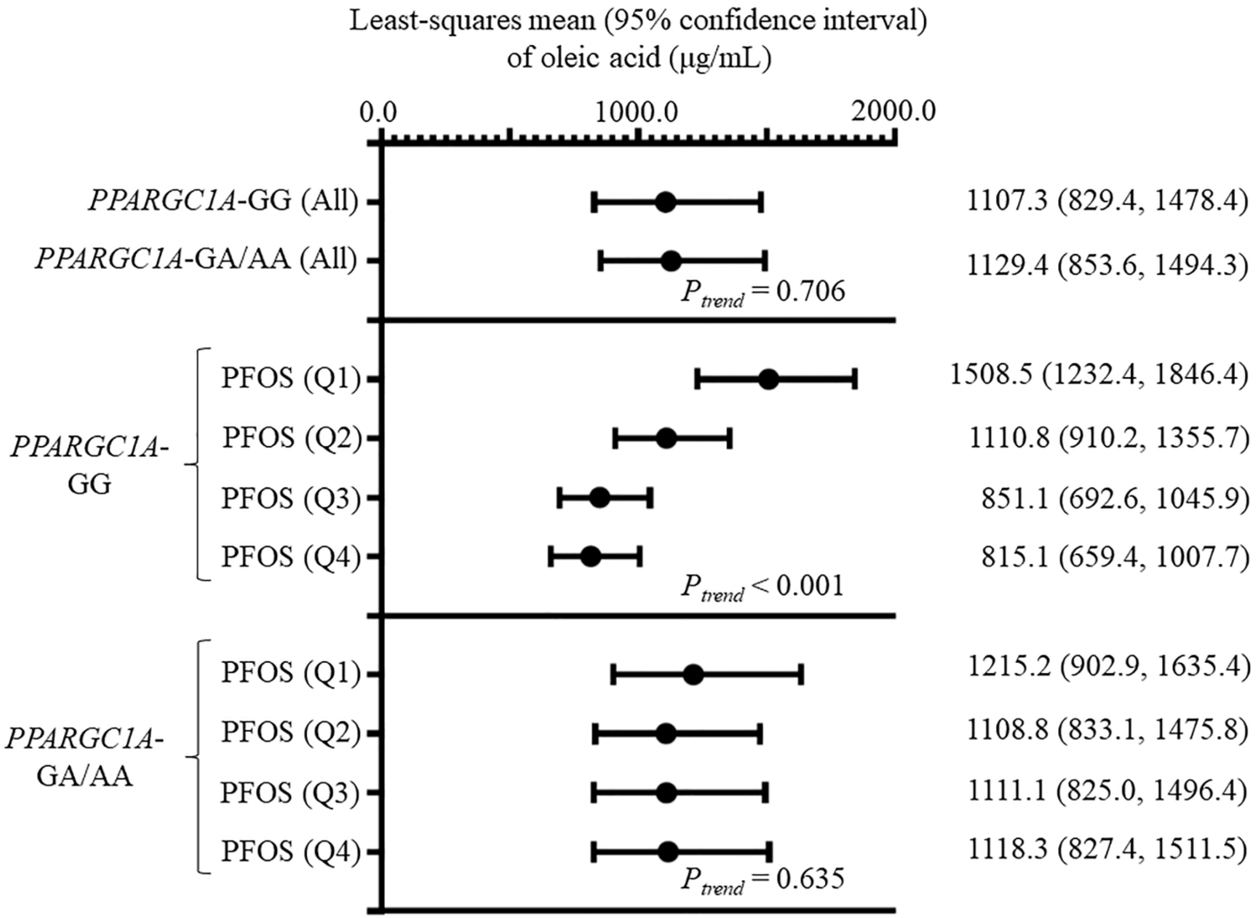

Supplementary Fig. S1 (D)

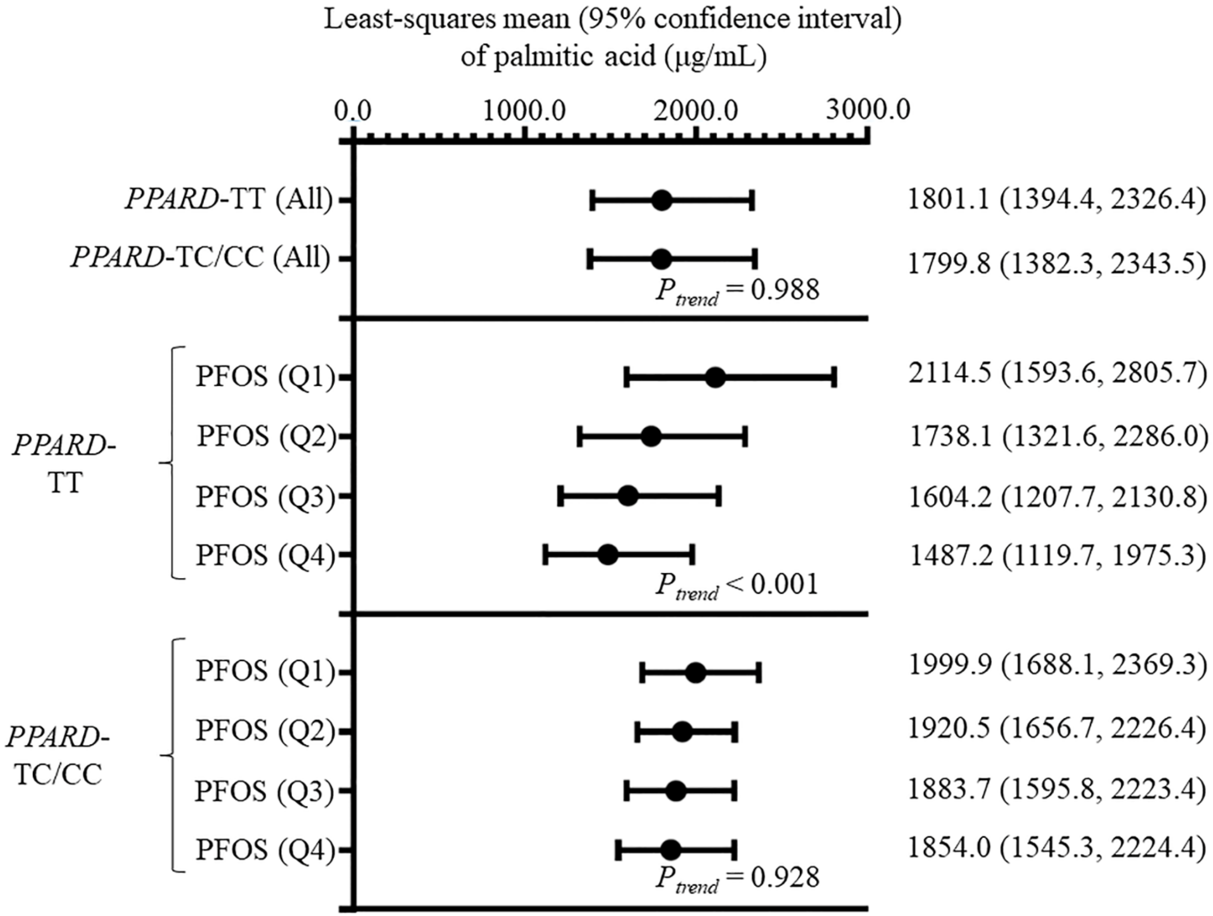

Supplementary Fig. S1 (E)

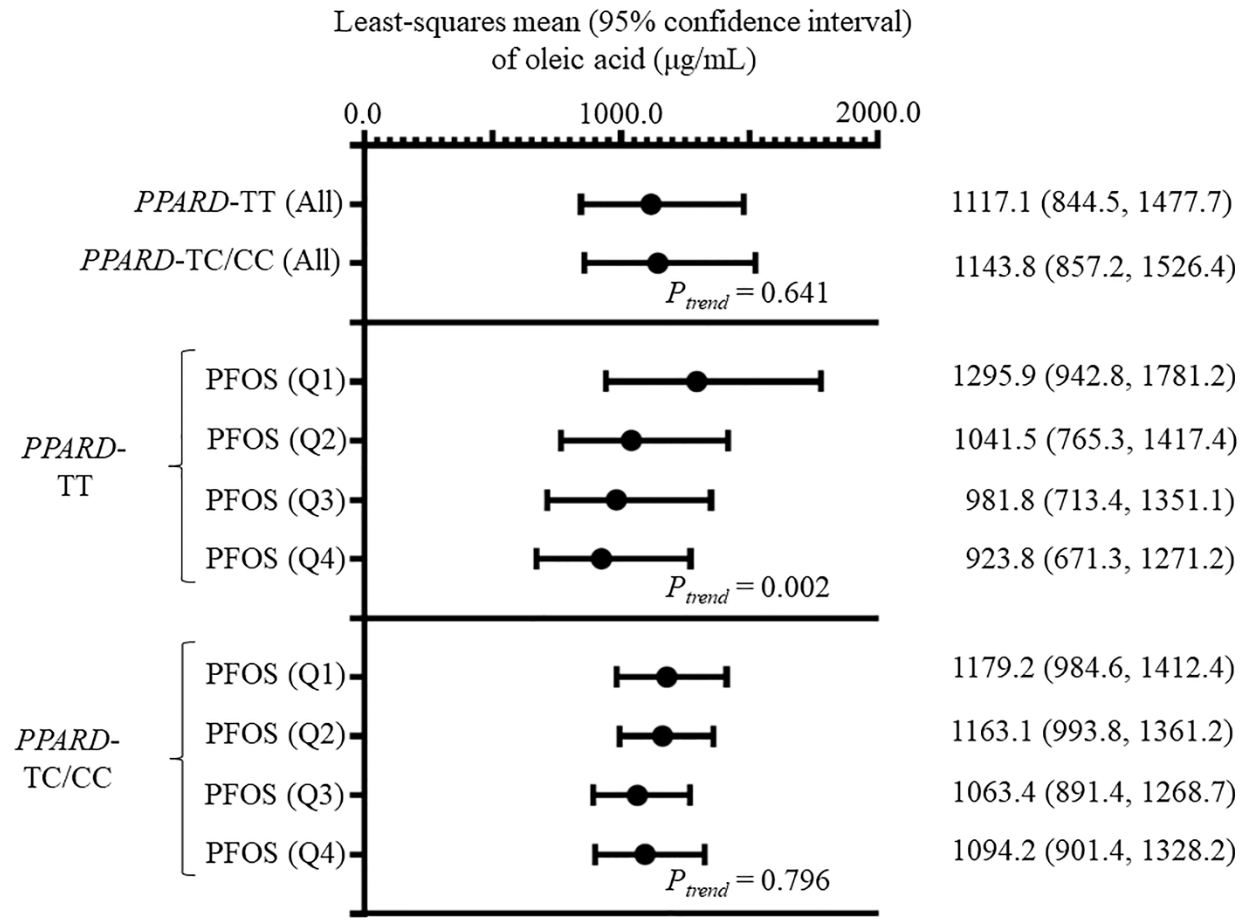

Supplementary Fig. S1 (F)

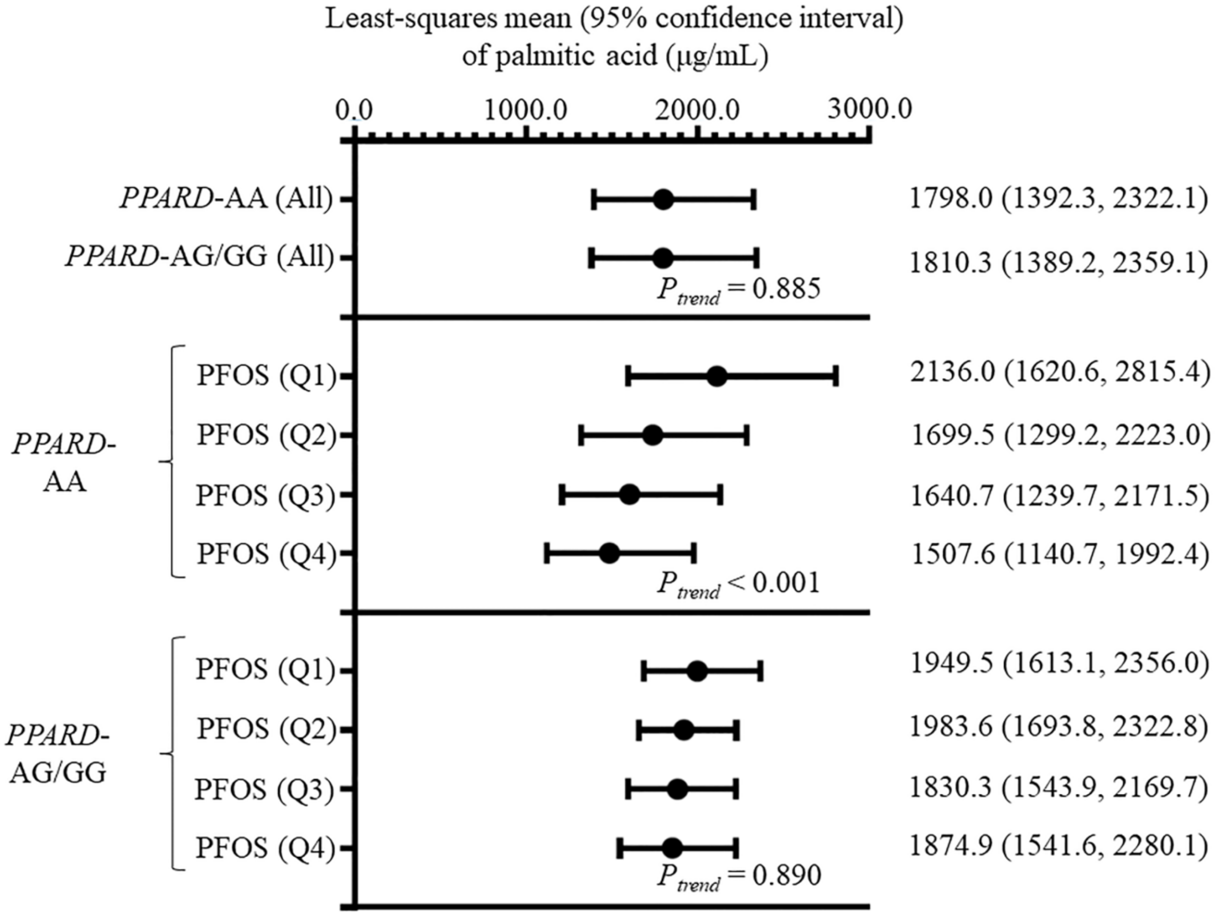

Supplementary Fig. S1 (G)

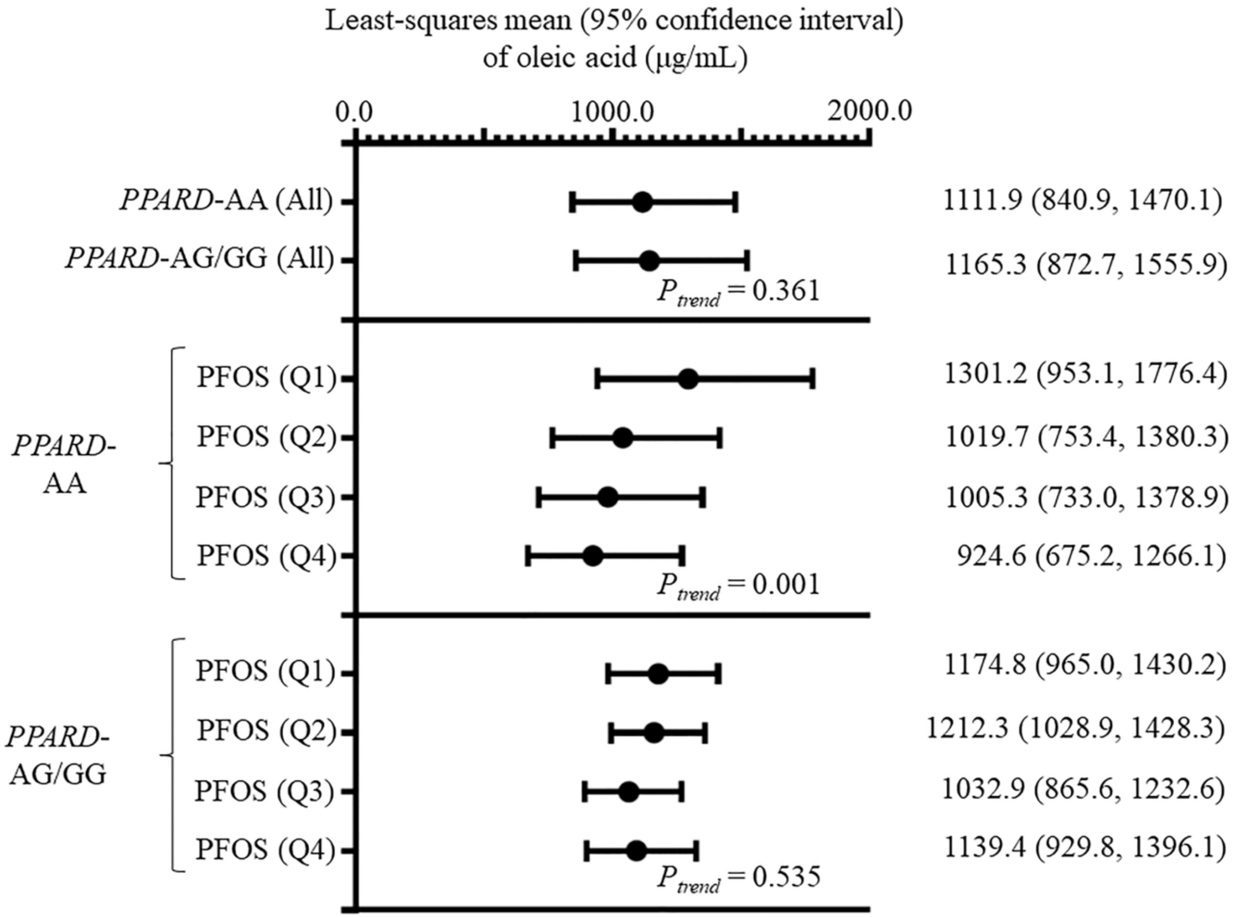

Supplementary Fig. S1 (H)
